# Seroepidemiological study of factors affecting anti-spike IgG antibody titers after a two-dose mRNA COVID-19 vaccination in 3744 healthy Japanese volunteers

**DOI:** 10.1038/s41598-022-20747-x

**Published:** 2022-09-29

**Authors:** Aya Sugiyama, Akemi Kurisu, Shintaro Nagashima, Kiyomi Hando, Khilola Saipova, Sayyora Akhmedova, Kanon Abe, Hirohito Imada, Md Razeen Ashraf Hussain, Serge Ouoba, Bunthen E, Ko Ko, Tomoyuki Akita, Shinichi Yamazaki, Michiya Yokozaki, Junko Tanaka

**Affiliations:** 1 Department of Epidemiology, Infectious Disease Control and Prevention, Graduate School of Biomedical and Health Sciences, Hiroshima University, 1-2-3, Kasumi, Minami-ku, Hiroshima, 734-8551 Japan; 2 Department of Clinical Radiology and Oncology, Andijan State Medical Institute, Andijan, Uzbekistan; 3 Department of Cardiorheumatology, Republican Specialized Scientific-Practical Medical Center of Pediatrics, Tashkent, Uzbekistan; 4 Unité de Recherche Clinique de Nanoro (URCN), Institut de Recherche en Science de La Santé (IRSS), Nanoro, Burkina Faso; 5 Payment Certification Agency (PCA), Ministry of Health, Phnom Penh, Cambodia; 6 Division of Clinical Laboratory Medicine, Hiroshima University Hospital, Hiroshima, Japan

**Keywords:** Viral infection, Preventive medicine, Epidemiology

## Abstract

Several factors related to anti-spike(S) IgG antibody titers after mRNA COVID-19 vaccination have been elucidated, but the magnitude of the effects of each factor has not been fully understood. This cross-sectional study assessed anti-S and anti-nucleocapsid (N) antibody titers on 3744 healthy volunteers (median age, 36 years; IQR, 24–49 years; females, 59.0%) who received two doses of mRNA-1273 or BNT162b2 vaccine and completed a survey questionnaire. Multiple regression was conducted to identify factors associated with antibody titers. All but one participant tested positive for anti-S antibodies (99.97%). The following factors were independently and significantly associated with high antibody titer: < 3 months from vaccination (ratio of means 4.41); mRNA-1273 vaccine (1.90, vs BNT162b2); anti-N antibody positivity (1.62); age (10’s: 1.50, 20’s: 1.37, 30’s: 1.26, 40’s: 1.16, 50’s: 1.15, vs ≧60’s); female (1.07); immunosuppressive therapy (0.54); current smoking (0.85); and current drinking (0.96). The largest impact on anti-S IgG antibody titers was found in elapsed time after vaccination, followed by vaccine brand, immunosuppressants, previous SARS-CoV-2 infection (anti-N antibody positive), and age. Although the influence of adverse reactions after the vaccine, gender, smoking, and drinking was relatively small, they were independently related factors.

## Introduction

In December 2019, an outbreak of pneumonia associated with severe acute respiratory coronavirus 2 (SARS-CoV-2) was reported in Wuhan, China. It spread throughout the world in several weeks. The World Health Organization (WHO) declared on 11 March 2020 a global pandemic of the novel coronavirus disease (COVID-19). As of 31 March 2022, there were a total of 485,243,022 confirmed cases worldwide, including 6,137,553 deaths^1^. In Japan, 6,538,890 confirmed cases have been reported, including 28,089 deaths^2^. As a result of worldwide efforts, several COVID-19 vaccines began to roll out around the end of 2020. Between February and March 2021, Japanese drug authorities approved two messenger RNA (mRNA)-based vaccine products: BNT162b2 (Pfizer-BioNTech) and mRNA-1273 (Moderna). It is reported that 79.5% of the Japanese population have completed two doses of either vaccine at the time of writing^3^. These vaccines were reported to be approximately 95% effective in preventing COVID-19 infection^4–6^.

Several factors related to antibody titers after mRNA COVID-19 vaccination have been elucidated, such as the time elapsed since the last vaccination^7–13^, age^14–16^, underlying conditions^17–24^, history of SARS-CoV-2 infection^25–30^, and vaccine brand^31^. It is reported that mRNA vaccines cause a high incidence of adverse reactions^4,6,15,32,33^, but the relationship between adverse reactions and antibody responses has not yet reached a consensual conclusion^14,16,34–36^. Although some possible factors are known to be related to antibody titers, analysis by adjusting potential confounders to clarify the magnitude of their effects is still needed.

We recruited a large cohort of healthy volunteers in Hiroshima, Japan, who received two mRNA vaccine doses and evaluated their serum antibody levels. The main purpose of this study was to investigate the clinical determinants of anti-spike protein (S) antibody titers following two doses of mRNA COVID-19 vaccine.

## Methods

### Study subjects

This was a large cross-sectional study of the general population conducted between August and September 2021. We identified a total of 21,406 individuals at Hiroshima University, consisting of 6059 staff members and 15,347 students. We sent them an e-mail asking for their willingness to undergo a serological test for anti–SARS-CoV-2 antibodies. Agreement and informed consent were obtained from 3915 persons, and their blood was collected to measure anti-SARS-CoV-2 antibody titers. In addition, a self-administered questionnaire survey was also conducted. The questionnaire had 17 items, including age, sex, other demographic factors, vaccination history, adverse reactions after vaccination, symptoms within the previous six months, personal infection prevention measures taken, history of COVID-19 infection or close contact with COVID-19 patients, social behavior patterns, comorbidities (hypertension, diabetes mellitus, heart disease, stroke, chronic obstructive lung disease, asthma, renal failure requiring dialysis, cancer, anemia, immunosuppression), smoking status, and drinking status. Overall, 3744 persons received two doses of mRNA-1273 or BNT162b2 vaccine and fully completed the questionnaire.

### Laboratory assays

Venous blood samples (10 mL) were collected at the survey site and sent to the analytical laboratory at Hiroshima University the same day. Serum aliquots were prepared and stored at − 20 degrees until serological analysis. Two U.S Food and Drug Administration (FDA) approved immunoassays were used to measure the titers of anti-spike protein (S) antibody immunoglobulin G (IgG) and anti-nucleocapsid protein (N) IgG.

VITROS Anti–SARS-CoV-2 S1 Quant IgG (Ortho Clinical Diagnostics; Rochester, NY, United States, sensitivity: 94.0%, specificity 100%^37^ was used to detect Anti-S IgG on the VITROS 3600 Immunodiagnostic System (Ortho Clinical Diagnostics; Rochester, NY, United States). This chemiluminescent enzyme immunoassay targets the SARS-CoV-2 S protein, allowing the detection of antibodies due to vaccination or natural infection. The WHO standard binding antibody unit/mL (BAU/mL) was used, and ≥ 17.8 BAU/mL was defined as positive^38^. The upper limit of measurement was 4000 BAU/mL.

SARS-CoV-2 N IgG was measured using the Elecsys Anti-SARS-CoV-2 (Roche Diagnostics International Ltd.; Rotkreuz, Switzerland, sensitivity: 99.5%, specificity 99.8%^39^, a qualitative electrochemiluminescent immunoassay performed on the Cobas 8000 analyzer series e801 (Roche Diagnostics International Ltd.; Rotkreuz, Switzerland). This test returns a positive result (cutoff index ≥ 1.0) only in subjects with a history of natural infection.

All tests were performed in accordance with the manufacturers’ instructions, and calibrations were inspected before analysis.

### Ethics approval and consent to participate

The ethics committee for epidemiological research of Hiroshima University approved this study (approval number, E-2123-2). Informed consent was obtained from all participants before any study procedures. All study activities were performed in accordance with the Declaration of Helsinki and relevant guidelines and regulations in Japan.

### Statistical analysis

Categorical variables are reported as numbers (percentages) and continuous variables as median and interquartile range (IQR). The Wilcoxon test was used to compare antibody titers in two groups, and the Kruskal–Wallis test was employed for more than two groups. Multivariate linear regression was used to assess associations between log-transformed IgG antibody titers and the following variables: gender, age, previous SARS-CoV-2 infection (defined as anti-N antibody positivity), vaccine brand, number of months since the second dose of vaccine, post-vaccination symptoms (severity was classified into three levels: away from work for more than two days, not away from work for more than two days, none), smoking status, drinking status, and presence of comorbidities. However, regarding comorbidities, heart disease, stroke, chronic obstructive lung disease, renal failure requiring dialysis, and cancer were found in less than 20 participants and were excluded from the analysis. Selected variables by stepwise procedure (P < 0.25) were used for multivariate analysis. All *p-*values were two-sided, and *p-*values < 0.05 were considered statistically significant. Data analysis was performed using JMP Pro 16.0 software (SAS Institute Inc., Cary, NC, USA).

## Results

### Characteristics of the participants

**Figure 1:**
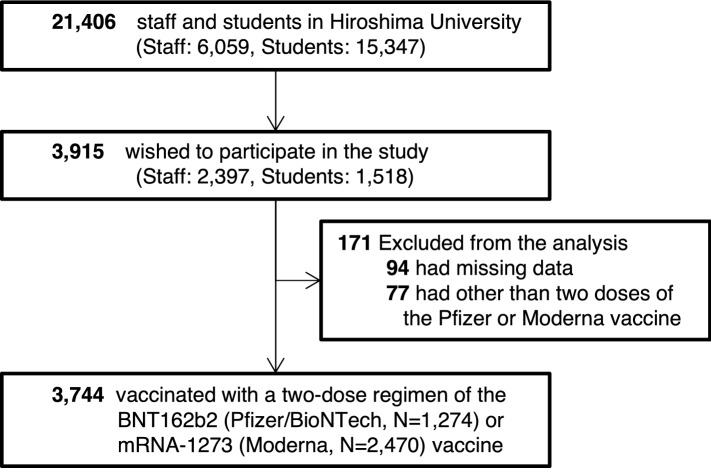
Study population of vaccinated healthy volunteers.

Among the 21,406 individuals who received our recruitment e-mail, 3915 agreed to participate (18.3%). Of these, 3744 had received two vaccine doses and completed the questionnaire fully (Fig. 1). Table 1 summarizes the demographic and background characteristics of the study participants. The median age was 36 years (IQR, 24–49 years), and females accounted for 59.0% of the participants. By vaccine brand, mRNA-1273 was more common (66.0%) than BNT162b2 (34.0%). The median number of days from the second vaccination to blood collection was 37 (IQR, 29–60) (Supplementary Fig. S1). Due to post-vaccination adverse reactions, 18.6% had been away from work or school for more than two days, whereas 76.7% had adverse reactions but did not miss work or school for over two days, and 4.7% had no adverse reactions. The most common adverse reactions were severe injection site soreness (73.6%), fever (temperature ≥ 37.5 °C) (68.7%), fatigue (67.5%), headache (49.9%), and joint and muscle pain (47.3%).

**Table 1:** Demographic characteristics of the study participants (N = 3744).

Characteristic	N	%
**Sex**		
Female	2209	59.0
Male	1535	41.0
**Age, median (IQR), year**	36 (24–49)	
**Age group, year**		
10–19	147	3.9
20–29	1331	35.6
30–39	605	16.2
40–49	733	19.6
50–59	632	16.9
≥ 60	296	7.9
**Previous SARS-CoV-2 infection**^**a**^		
Yes (anti-N antibody positive)	32	0.9
No (anti-N antibody negative)	3712	99.1
**Vaccine brand**		
mRNA-1273 (Moderna)	2470	66.0
BNT162b2 (Pfizer/BioNTech)	1274	34.0
**Time since the second dose, day, median (IQR)**	37 (29–60)	
**Time since the second dose, month**		
< 3	2896	77.4
≥ 3	848	22.7
**Post-vaccination symptoms (any)**		
Yes, away from work for more than 2 days	697	18.6
Yes, but not away from work for more than 2 days	2870	76.7
None	177	4.7
**Post-vaccination symptoms**		
Severe injection site soreness	2755	73.6
Fever (temperature ≥ 37.5 °C)	2571	68.7
Fatigue	2529	67.5
Headache	1867	49.9
Joint and muscle pain	1771	47.3
Nausea	289	7.7
Diarrhea	197	5.3
Skin rash	143	3.8
**Current smoking**		
Yes	162	4.3
No	3582	95.7
**Current drinking**		
Yes	1602	42.8
No	2142	57.2
**Comorbidities**		
Hypertension	218	5.8
Diabetes	57	1.5
Heart disease	13	0.3
Stroke	8	0.2
Chronic obstructive lung disease	3	0.1
Asthma	146	3.9
Renal failure requiring dialysis	2	0.1
Cancer	13	0.3
Anemia	57	1.5
Immunosuppression	24	0.6

*SAR-CoV-2* severe acute respiratory syndrome coronavirus 2.

^a^Previous SARS-CoV-2 infection was defined anti-SARS-CoV-2 Nucleocapsid (N) IgG antibody positivity.

### Anti-S and anti-N IgG antibody titers

After two doses of BNT162b2 or mRNA-1273 vaccine, all recipients were positive for anti-S IgG except one person (3743/3744; 99.97%; 95% confidence interval (CI), 99.92–100%). Thirty-two participants also tested positive for anti-N IgG antibodies, suggesting previous SARS-CoV-2 infection (Table 2). Regarding the question “Were you infected with SARS-CoV-2 in the past?”, 9 of these 32 participants (28.1%) responded "yes", 19 (59.4%) responded "no", and 4 (12.5%) responded "I do not know”.

**Table 2:** Anti-Spike (S) IgG and anti-Nucleocapsid (N) IgG titers after 2 doses of mRNA-1273 or BNT162b2 COVID-19 vaccine in the general population (N = 3744).

	Anti-nucleocapsid (N) IgG		
	Negative	Positive	Total
Anti-spike (S) IgG*			
Negative	1 (0.03%)	0 (0.00%)	1 (0.03%)
Positive	3711 (99.12%)	32 (0.85%)	3743 (99.97%)
Total	3712 (99.15%)	32 (0.85%)	3744 (100%)

Anti-spike (S) IgG was measured using Vitros Anti–SARS-CoV-2 S1 Quant IgG (Ortho Clinical Diagnostics). Positive was defined as ≥ 17.8 binding antibody units/mL.

Anti-nucleocapsid (N) IgG was measured using Elecsys Anti-SARS-CoV-2 (Roche Diagnostics International Ltd.). A positive result was defined as cutoff index ≥ 1.0.

The participant who tested negative for both anti-S and anti-N antibodies was a male in his 60 s, with autoimmune disease, immunosuppressive therapy, hypertension, diabetes mellitus, chronic kidney disease, and a history of stroke. His blood was collected one month after receiving the second dose of the mRNA-1273 vaccine.

### Anti-S IgG antibody titers

The median anti-S IgG antibody titer was 1750 BAU/mL (IQR, 651.3–3197.5). Supplementary Fig. S2 shows the distribution of anti-S IgG antibody titers by sex, age, anti-N antibody positivity, vaccine brand, time since the second dose, post-vaccination symptoms, smoking status, drinking status, and current history of hypertension, diabetes, anemia, asthma, immunosuppressive therapy, heart disease, cancer, and stroke.

### Clinical determinants of anti-S IgG antibody titers after two vaccine doses

**Figure 2:**
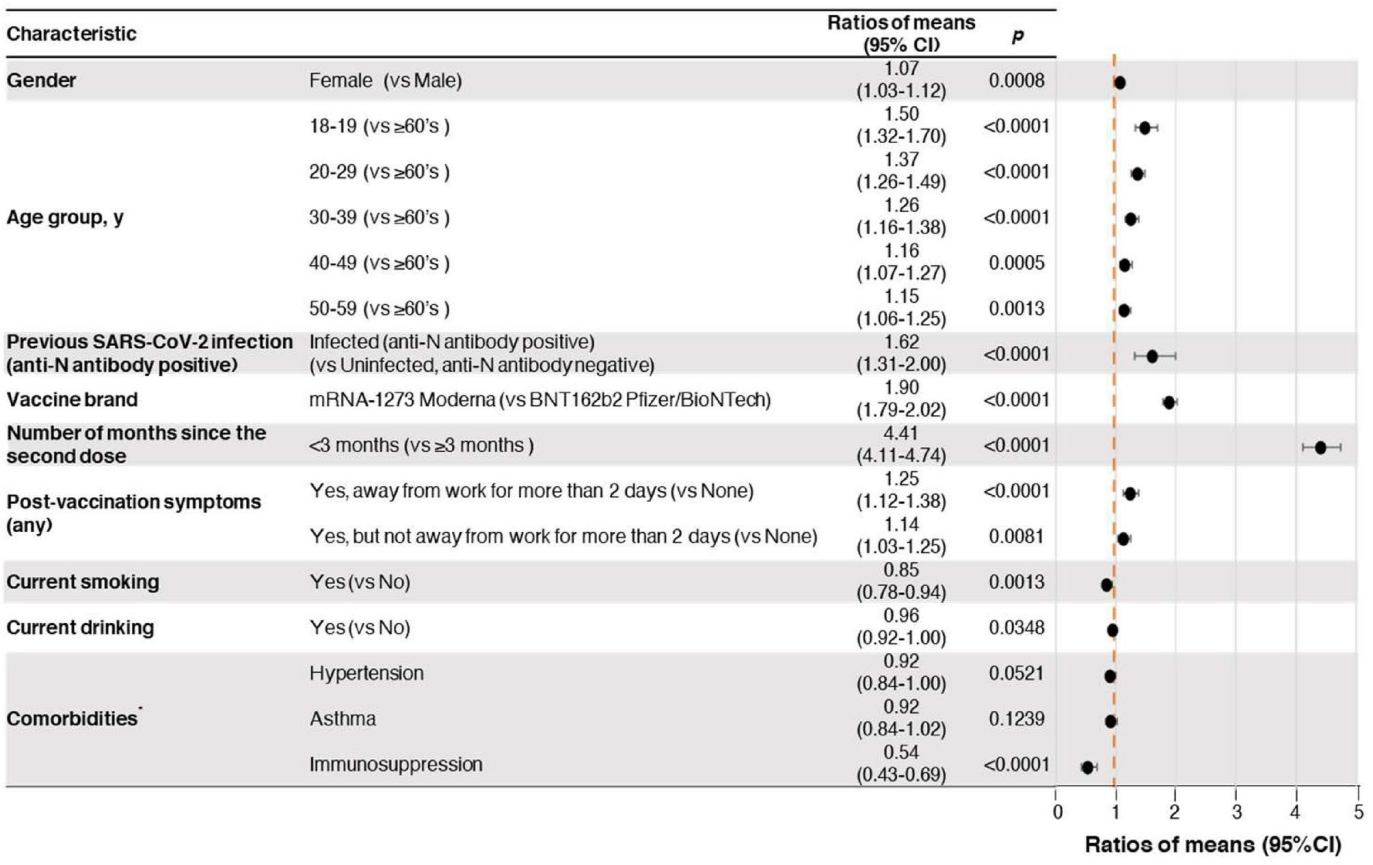
Multivariate linear regression results for anti-spike (S) protein IgG antibody titers after the second dose of mRNA COVID-19 vaccine. Model *P* < 0.0001, R^2^ = 0.7542. *Comorbidities with fewer than 20 participants (heart disease, stroke, chronic obstructive lung disease, renal failure requiring dialysis, cancer) were excluded from the analysis. Variables were selected using the stepwise method (*P* < 0.25). *SARS-CoV-2* severe acute respiratory syndrome coronavirus 2, *RoM* ratios of means, *CI* confidence interval, *IgG* immunoglobulin G.

Multivariate linear regression identified the following factors as independently and significantly associated with anti-S IgG antibody titers after the second vaccine dose (Fig. 2): < 3 months after vaccination (ratio of means (RoM), 4.41; 95% CI 4.11–4.74); mRNA-1273 vaccine (vs. BNT162b2 vaccine; RoM, 1.90; 95% CI 1.79–2.02); anti-N antibody positivity (RoM, 1.62; 95% CI 1.31–2.00); age (reference group: ≥ 60 years; 10–19 years: RoM, 1.50; 95% CI 1.32–1.70; 20–29 years: RoM, 1.37; 95% CI 1.26–1.49; 30–39 years: RoM, 1.26; 95% CI 1.16–1.38; 40–49 years: RoM, 1.16; 95% CI 1.07–1.27; 50–59 years: RoM, 1.15; 95% CI 1.06–1.25); adverse reaction to vaccine (requiring ≥ 2 days leave from work or school vs. no adverse events: RoM, 1.25; 95% CI 1.12–1.38; not requiring for ≥ 2 days leave from work or school despite symptoms vs. no adverse events: RoM, 1.14, 95% CI 1.03–1.25); female sex (RoM, 1.07; 95% CI 0.13–1.12); current smoking (RoM, 0.85; 95% CI 0.78–0.94); current drinking (RoM, 0.96; 95% CI 0.92–1.00); and current immunosuppressive therapy (RoM, 0.54; 95% CI 0.43–0.69). Diabetes and anemia were not selected by the stepwise method.

## Discussion

This cross-sectional study of a large cohort of healthy volunteers provided seroepidemiological data on anti-S IgG antibody titers and the factors that influenced them after two doses of mRNA-1273 or BNT162b2 vaccine. The major strength of this study is its large sample size which allowed for a reliable multivariate analysis. The most significant impact on anti-S IgG antibody titers after two doses of mRNA COVID-19 vaccine was found in elapsed time after vaccination, followed by vaccine brand, immunosuppressants, previous SARS-CoV-2 infection (anti-N antibody positive), and age. Although the influence of adverse reactions after the vaccine, gender, smoking, and drinking was relatively small, they were independently related factors. There have been many reports on post-vaccination antibody titers attenuation over time^7–13,26,40^. The results of this study revealed that the elapsed time after vaccination most affected the anti-S IgG antibody titer after adjusting with other related factors. So far, no vaccinated person experienced anti-S IgG seroreversion after seven months of follow-up^26^, but further long-term monitoring and research on the status of anti–SARS-CoV-2 antibodies is warranted.

After adjusting for other relevant factors, this study showed that the mean anti-S antibody titer was 1.9 times higher in those who received the mRNA-1273 vaccine (Moderna) compared to those who received the BNT162b2 vaccine (Pfizer/BioNTech). Previous studies have also demonstrated a significantly higher humoral immunogenicity and slower decay rate of antibody titer in those who received the mRNA-1273 vaccine compared with the BNT162b2 vaccine^31,41,42^. There are two possible explanations for this difference. First, compared to the BNT162b2 vaccine, the mRNA-1273 vaccine has approximately three times the amount of mRNA material, resulting in higher antibody titers^31^. Second, there are some reports that the antibody titer is higher when the period between the first and the second dose is longer^43–45^. The interval between the first and second doses is one week longer for the mRNA-1273 vaccine compared to the BNT162b2 vaccine (4 weeks vs. 3 weeks). On the other hand, the mRNA-1273 vaccine is also known to have a higher frequency of adverse reactions than BNT162b2^32^.

In general, vaccine-induced immunity is largely influenced by genetics, environmental factors, nutrition, sex, and the microbiome^46^. Because of hormonal, genetic, and microbiota differences, females typically develop higher antibody titers and experience more adverse events following vaccination than males^46,47^. Elderly individuals have lower antibody titers due to compromised T cell and plasma cell function^14–17,48^. Our study also showed that female sex and young age are both predictors of stronger antibody responses, but the impact of sex was relatively small. Unfortunately, this study could not examine people over 75 years, and further studies are required to address this age group. As for immunosuppressive therapy, it was significantly associated with lower antibody titers, as in previous reports^18,19,22–24,49^, because patients with immunosuppression have poor humoral and cellular immune responses^49^.

In this study, current smoking and current drinking were associated with lower antibody titers. These findings were consistent with previous studies^50,51^, suggesting that these lifestyle habits compromise immune function.

In individuals with normal immune function, SARS-CoV-2 infection induces SARS-CoV-2–specific memory cells, which produce anti-S antibodies up to 6 months after infection^52^. Post-vaccination anti-S IgG antibody titers are higher in patients with a history of natural infection than those without^25,27–30,53^. Our study also showed that previous SARS-CoV-2 infection is significantly associated with higher antibody levels.

Adverse reactions after mRNA-based COVID-19 vaccination are common^4,6,15,32,33^. Previous study results were inconsistent regarding the association between adverse reactions and antibody titers^14,34–36,54^ . We found a tendency for anti-S IgG antibody titers to be high when adverse reactions were severe, but the impact was relatively small after adjusting for multiple confounding factors. The causal relationship between adverse reactions after mRNA-based COVID-19 vaccination and antibody reactions has not been fully elucidated. Type I interferon, multiple pro-inflammatory cytokines, and chemokines produced by the booster vaccine, which induce injection-site and systemic inflammation (side effects), are supposed to promote an antibody-producing immune response^55^. However, all study participants who did not experience adverse reactions still acquired anti-S antibodies, suggesting that the absence of adverse reactions does not rule out immunogenicity. This finding supports the need to improve vaccine safety and reduce the rate of adverse reactions.

During the study period (August–September 2021), Hiroshima Prefecture was in the midst of the fifth epidemic wave of infections with the alpha and delta variants. COVID-19 cases confirmed by the end of August 2021 accounted for 0.67% of the total population in Hiroshima (approximately 2.8 million)^56^. Elevated anti-N IgG titers are a hallmark of past natural SARS-CoV-2 infection. We determined that 0.85% of study participants had a history of SARS-CoV-2 infection, as evidenced by anti-N IgG testing. Among them, 71.9% were not aware of a previous infection. Since asymptomatic patients accounted for 16–33.3% of COVID-19 patients in previous studies^57–59^, there was a much higher prevalence of asymptomatic SARS-CoV-2 infection in this study. This might be partly attributable to our study participants being relatively young (median age, 36 years). Also, although the anti-N IgG testing is highly specific, false positive results due to nonspecific reactions may be possible because this study was conducted in a population with a very low prevalence of past infections^39,60^.

Our study has several limitations. First, the study participants consisted of staff members and students of the university community. This population could have been skewed towards the healthier and younger ends of the general population. Our study did not include individuals aged 75 years or older because of the retirement age regulations; further investigations in this age group are warranted. The results of this study should be interpreted as representative of the healthy general population, and the characterization of post-vaccination antibodies in populations with specific comorbidities should be revealed by other studies. In addition, study participants were mostly individuals of Japanese origin, which did not allow for comparisons by race or ethnicity. Second, comorbidities and adverse reactions were self-reported by participants and could be a source of recall bias and underestimation. Third, this study did not investigate the effects of neutralizing antibodies and T-cell responses. Even though a strong correlation has been reported between neutralizing antibody titers and IgG titers^17,40,61,62^, the relationship between antibody titers and the clinical protection potential of vaccines, as well as their effects on new variants, is not sufficiently clear.

## Conclusions

This study clarified factors influencing anti-S IgG antibody titers after two mRNA vaccine doses based on a large set of seroepidemiological data. The results of this study may serve as a reference for the third and subsequent vaccine policy developments in a general population: the people who should be most prioritized for the third vaccination are those with a long time since the second vaccination. Priority should then be given to those who received BNT162b2 vaccine instead of mRNA-1273, followed by those on immunosuppressants, those without past infection (including asymptomatic infections), and people aged 60 years or older. Finally, the booster dose should also be advised to people who had no adverse reactions after vaccination, males, smokers, and alcohol consumers.

## Supplementary Information

Supplementary Information 1.

Supplementary Information 2.

## Data Availability

The datasets used and analyzed during the current study are available from the corresponding author on reasonable request.
