# Supplementary figures and images for: Seroepidemiological study of factors affecting anti-spike IgG antibody titers after a two-dose mRNA COVID-19 vaccination in 3744 healthy Japanese volunteers
Source: Sci Rep. 2022 Sep 29;12:16294. doi: 10.1038/s41598-022-20747-x (PMC9520958; doi:10.1038/s41598-022-20747-x)

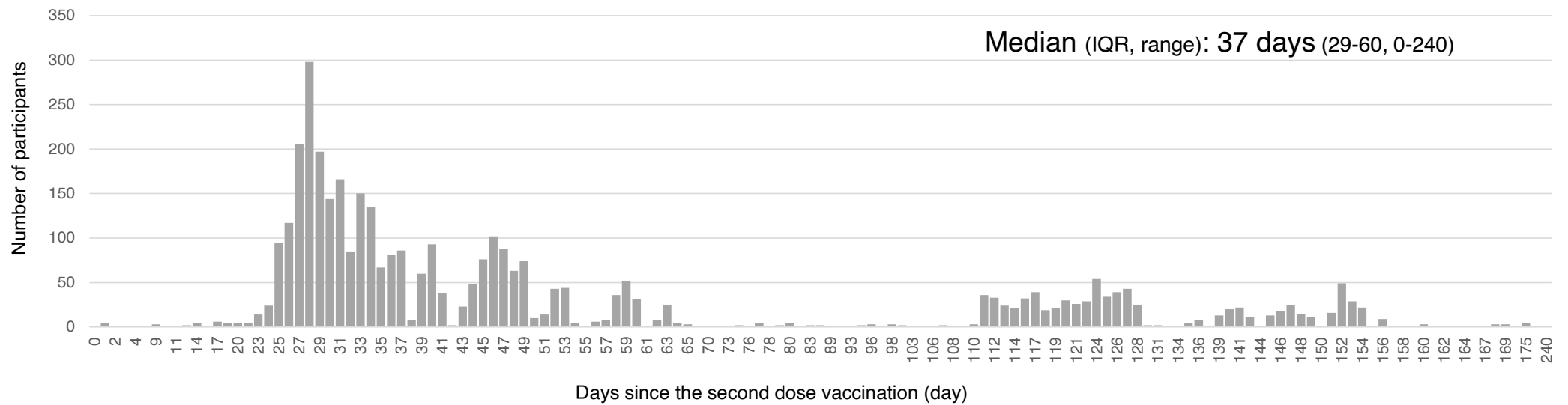

Supplementary figure 1. Distribution of days since the second dose in 3,744 participants

Supplement: Supplementary file 1 — Supplementary Information 1. [file 41598_2022_20747_MOESM1_ESM.pdf]
