# Supplementary material for: Seroepidemiological study of factors affecting anti-spike IgG antibody titers after a two-dose mRNA COVID-19 vaccination in 3744 healthy Japanese volunteers
Source: Sci Rep. 2022 Sep 29;12:16294. doi: 10.1038/s41598-022-20747-x (PMC9520958; doi:10.1038/s41598-022-20747-x)
Supplement: Supplementary file 2 — Supplementary Information 2. [file 41598_2022_20747_MOESM2_ESM.pdf]

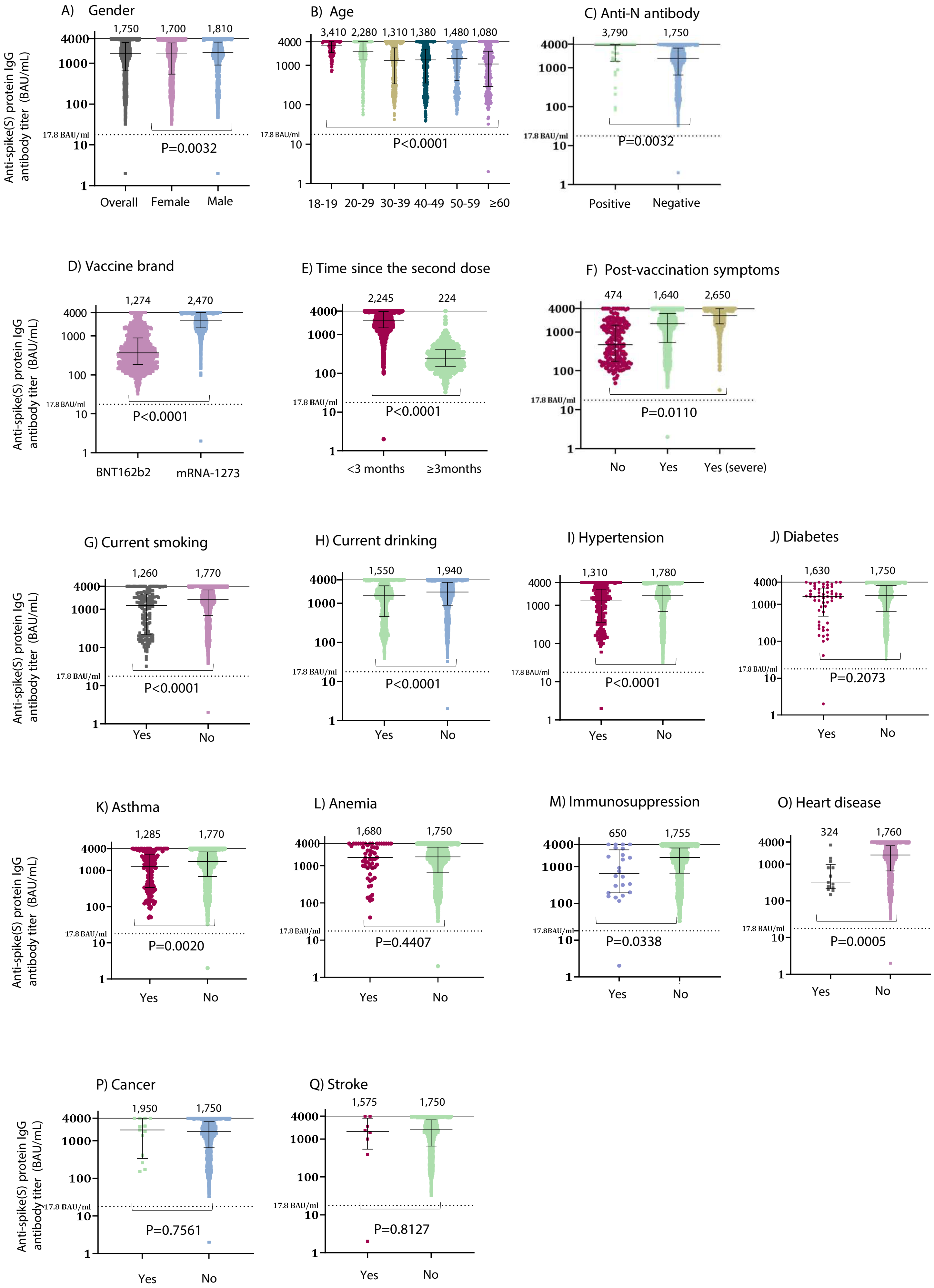

Supplementary figure 2. Anti-spike (S) protein IgG antibody titers in 3,744 healthy volunteers after the second dose of mRNA COVID-19 vaccine
