# Supplementary material for: Author Correction: Seroepidemiological study of factors affecting anti-spike IgG antibody titers after a two-dose mRNA COVID-19 vaccination in 3744 healthy Japanese volunteers
Source: Sci Rep. 2023 Feb 2;13:1906. doi: 10.1038/s41598-023-28884-7 (PMC9893206; doi:10.1038/s41598-023-28884-7)
Supplement: Supplementary file 1 — Supplementary Information 2. [file 41598_2023_28884_MOESM1_ESM.pdf]

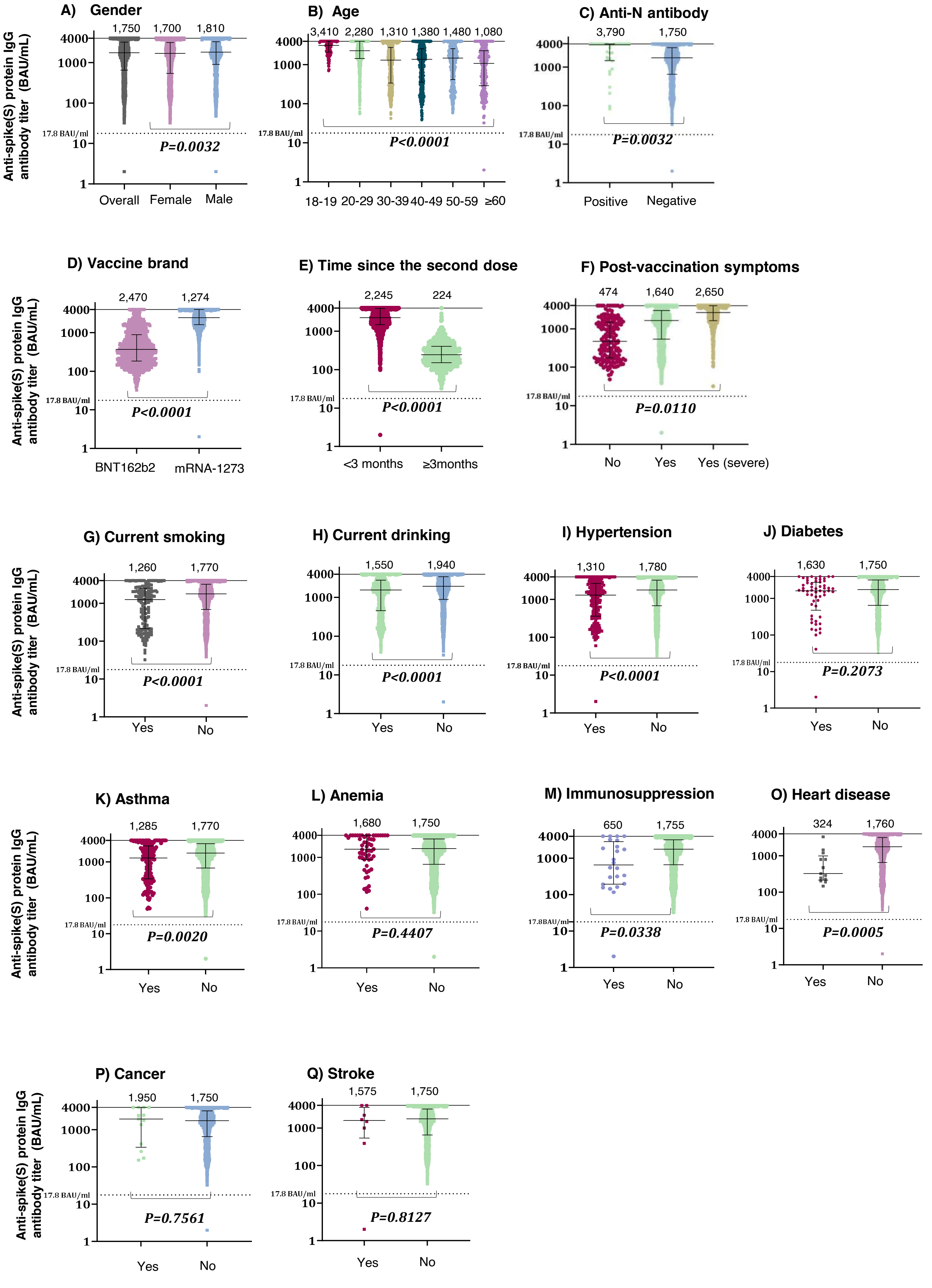

Supplementary figure 2. Anti-spike (S) protein IgG antibody titers in 3,744 healthy volunteers after the second dose of mRNA COVID-19 vaccine
